# Phenolic Compounds from Haskap Berries Have Structure, Combination, and Cell Line-Dependent Impacts on the Longevity-Associated Deacetylase Sirtuin 1

**DOI:** 10.3390/cells14040295

**Published:** 2025-02-17

**Authors:** Morgan A. Fleming, Nicholas H. Low, Christopher H. Eskiw

**Affiliations:** Department of Food and Bioproduct Sciences, University of Saskatchewan, Saskatoon, SK S7N 5A8, Canada; maf135@usask.ca (M.A.F.); nicholas.low@usask.ca (N.H.L.)

**Keywords:** phenolics, haskap berry, synergy, Sirtuin 1, longevity

## Abstract

It is well established that phenolic compounds from plant sources impact readouts of cell health such as reduced radical and reactive oxygen species. However, it is unclear if specific phenolic structures impact other cellular processes or proteins, such as the evolutionary conserved deacetylase Sirtuin 1 (SIRT1), and if phenolic combinations interact synergistically to do so. We observed that structurally diverse haskap berry phenolics (caffeic acid, cyanidin, kaempferol-3-*O*-glucoside, and gentisic acid) differentially impacted normal primary human fibroblast growth, which has been linked to SIRT1. These results were consistent with previous work from our lab indicating that haskap phenolic extracts/fractions impact human cell growth via SIRT1-dependent mechanisms. Therefore, we furthered the investigation into SIRT1 and phenolic structure and observed that the individual phenolics or their combinations had no observable impact on *SIRT1* transcript abundance or cellular localization. We also observed that select phenolics decreased SIRT1 protein abundance and increased SIRT1 activity. The catechol-containing phenolics outperformed those that lack a catechol group, indicating potential structure-dependent impact(s). Potential synergy between the specific phenolics analyzed was observed in Western blot, and potential antagonism was identified in the SIRT1 activity assay. Results were concomitant with the presence of different phenolic structures, phenolic combinations, and cell type (sex and/or individual differences). These results highlight the possible significance of the catechol structure and indicate that phenolics have the potential to impact cell processes, which the authors hypothesize to be due to mechanisms that are independent of antioxidant activity.

## 1. Introduction

Our environment has substantial impacts on readouts of cell health, with diet being one of the most significant factors. Furthermore, contrary to recent consumer trends like the carnivore diet, we have known for centuries that consuming an abundance of plants is known to promote increased health and lifespan (i.e., longevity) [1,2,3,4,5,6,7]. While there are many mechanisms by which plants can impact readouts of cell health, of great interest are a class of structurally diverse, naturally occurring, secondary metabolites called phenolic compounds (i.e., phenolics). Structurally, phenolics are classified as those compounds containing a six-carbon aromatic ring with at least one covalently bound hydroxyl group (i.e., phenolic ring). Phenolic compounds are postulated to have broad impacts on metabolic and biochemical processes, including but not limited to anti-aging, anticancer, anti-inflammatory, cardio and neuroprotective resulting from structure-dependent antioxidant activity [8,9,10,11,12]. Intriguingly, investigations into the antioxidant activity of phenolic combinations have demonstrated that specific phenolic combinations interact synergistically to increase antioxidant activity to a greater extent than that of the individuals [13,14,15,16]. However, the question remains unclear whether antioxidant activity is the only mechanism by which phenolics impact readouts of cell health. Due to the abundance of purported health benefits associated with phenolic consumption, it is logical that they have additional mechanisms to achieve these functions. Moreover, as there are over 10,000 structurally unique phenolics identified in nature, it is logical to predict that structurally different phenolics will have varied impacts on cellular proteins and pathways that regulate health, and that these different impacts can be explained structurally. Furthermore, as antioxidant synergy has been identified amongst phenolic combinations via in vitro chemical antioxidant assays, it follows that synergy could also occur in biological systems and this effect could be explained by phenolic structure. Recent research indicated that phenolics also promote longevity independent of antioxidant activity, via impacts on cellular pathways/processes such as the evolutionary conserved enzyme Sirtuin 1 (SIRT1) [17,18,19,20].

SIRT1 is a nicotinamide adenine dinucleotide (NAD^+^)-dependent deacetylase with established regulatory connections to longevity. Previous research has demonstrated that overexpression of SIRT1 in model organisms extended lifespan [21,22,23]. Furthermore, SIRT1 has established regulatory roles in processes that mediate cell stress [3,24,25,26]. As such, compounds that impact SIRT1 are of interest and notably, various phenolics have been shown to differentially impact SIRT1 [18,20,27,28]. Therefore, it is rational to predict that SIRT1 protein abundance and function are responsive to specific phenolic structures and/or combinations.

One of the most well-known phenolics that is known to impact SIRT1 is resveratrol, commonly recognized as a component of red grapes and wine. Although, the impacts of resveratrol on SIRT1 have been previously challenged, Huynh et al. (2022) [18] established that resveratrol directly interacts with SIRT1 allosterically to increase its activity and, therefore, promote longevity. Monceaux et al. (2022) [27] reported that the phenolics ferulic acid, pterostilbene, and tyrosol had SIRT1-dependent cardioprotective functions in H9c2 cells. Lopez-Fernandez-Sobrino et al. (2021) [28] reported that a phenolic-rich wine lees powder increased SIRT1 levels in hypertensive male rats. Notably, Zehfus et al. (2021) [20] reported that haskap berry phenolic fractions/extracts impact cellular stress sensing pathways in non-diseased (i.e., normal) primary and immortalized human fibroblasts (2DD and NB1hT, respectively). These impacts were shown to be SIRT1-dependent but achieved through indirect mechanisms. Importantly, this research demonstrated that different haskap phenolic extracts and fractions, created by solubilization in varying ethanol concentrations, achieved different effects on cell growth behavior and SIRT1 activity. Notably, when SIRT1 was knocked down using lentiviral transduction of siRNA, the observed impacts on cell growth were essentially abolished in NB1hT cells and greatly reduced in 2DD cells. The aforementioned results highlight that the majority of research on the connection between phenolics and SIRT1 focused on individual compounds or complex phenolic mixtures (fruit/vegetable extracts/fractions). These methods do not address the possibility that specific phenolic combinations have a greater impact than individuals (i.e., synergy). Moreover, a limitation of studying complex phenolic mixtures is the inability to determine which specific phenolic structures are/are not responsible for the observed function(s) or possibly contribute to synergy or antagony. Defining which phenolic structures can impact SIRT1 and do so synergistically can help us (1) define the mechanisms by which phenolics impact longevity; (2) identify which foods and/or supplements may function optimally (i.e., synergistically); and (3) rationally design nutraceuticals/pharmaceuticals to target SIRT1 and increase longevity.

**Figure 1:**
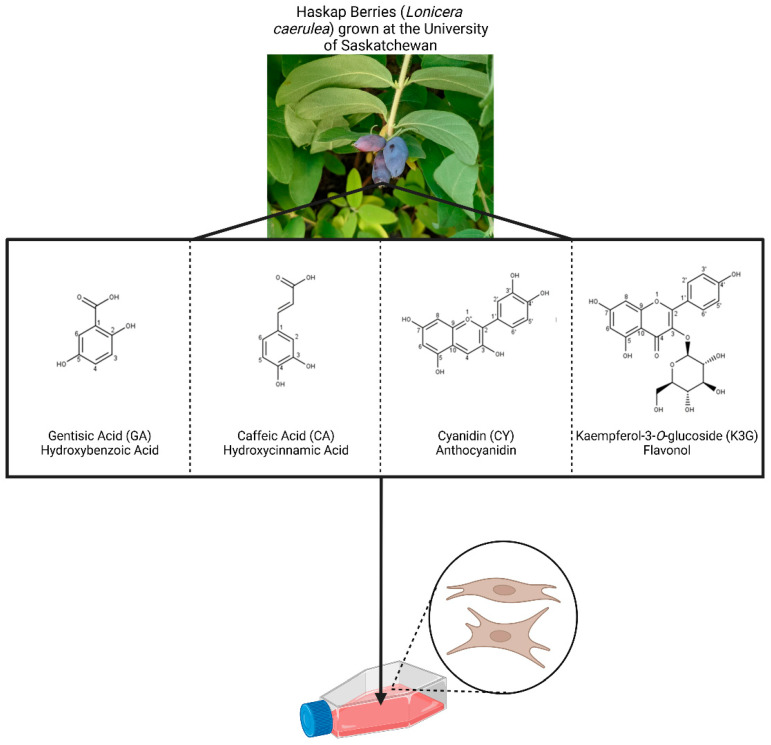
Haskap berries and the structures of the four phenolics selected for this work. The phenolics were supplemented to normal primary human fibroblasts individually to determine their function(s) and in equimolar combinations to examine synergy.

As discussed previously, complex haskap phenolic mixtures from Zehfus et al. [20] had impacts on human cells via SIRT1-dependent mechanisms. This work leads to the following new questions: (1) Do specific individual phenolics, and their associated structures, impact SIRT1 or are phenolic combinations acting synergistically to do so; and (2) at which regulatory level(s) do the phenolics impact SIRT1? The aim of the present research was to address these unknowns. Therefore, we selected four structurally different phenolics (Figure 1) to supplement individually and in combinations to normal primary human fibroblasts. The selected phenolics were the following: (1) caffeic acid (CA), a hydroxycinnamic acid; (2) cyanidin (CY), an anthocyanidin; (3) kampferol-3-*O*-glucoside (K3G), a flavonol; and (4) gentisic acid (GA), a hydroxybenzoic acid. Results indicated that CA and CY had larger impacts on SIRT1 and that the phenolics act primarily on SIRT1 protein abundance and activity, although these impacts were dependent on phenolic structure, combination, and cell line (sex and/or individual differences). Importantly, cells isolated from a human male (XY) and female (XX) were included in this work. While there are conserved mechanisms and phenotypes of aging and age-related disease, it is essential to explore and incorporate the inherent biological differences between sexes in tissue culture research.

## 2. Materials and Methods

### 2.1. Cell Culture

Two normal primary human fibroblasts were used in this research. The cells were isolated from a male (XY) newborn designated 2DD, and a 26-year-old female (XX), designated 07124B. The 07124B cells were purchased from Coriell.org and the ID number is AG07124. 2DD and 07124B cells were grown in Dulbecco’s Modified Eagle Medium (DMEM; 4.5 g/L D-glucose; pH 7.7) supplemented with 10% (*v*:*v*) fetal bovine serum (FBS), 0.1% (*v*:*v*) ciprofloxacin, and 1% (*v*:*v*) penicillin-streptomycin 10× solution (i.e., DMEM). Cells were seeded at an initial density of 3000 cells/cm^2^ and were not grown past 70% confluency to avoid contact inhibition. Cells were grown in a humidified incubator (Fisher Scientific, Marietta, OH, USA) at 37 °C with 5% carbon dioxide (CO_2_); with media replacement every 3–4 days. When cells grew to 70% confluency, they were harvested and reseeded (i.e., passaged); cells between passage 14–19 were used for this work. Passaging was performed by media aspiration followed by the addition of a predetermined volume of TrypLE Express (volume used was dependent on plate size). Cell dissociation from the surface of the plate was afforded by incubation at 37 °C with TrypLE Express for 5 min or until complete dissociation was visually observed. Cells were quantitatively transferred with media into 15 mL falcon tubes and were pelleted by centrifugation for 4 min at 174 relative centrifugal force (rcf; Centrifuge 5804 Eppendorf, Hamburg, Germany). The resulting pellet was resuspended in 10 mL of fresh DMEM, and a 10 µL aliquot of this suspension was mixed with 10 µL 0.4% trypan blue dye. The resulting mixture was incubated at 37 °C for 3 min and cells were counted employing a Countess II (Countess II FL, Thermo Fisher Scientific; Burlington, ON, Canada).

### 2.2. Cell Treatments

The four phenolics (caffeic acid [CA], cyanidin chloride [CY], kaempferol-3-*O*-glucoside [K3G], and gentisic acid [GA]) were individually dissolved in DMSO to prepare 30.0 mM stock solutions. Immediately prior to treatment, the stock solutions were diluted with DMEM to obtain a final phenolic concentration of 115.0 µM. This concentration was selected based on results from Zehfus and colleagues [20] who reported that a haskap phenolic fraction (40% ethanol) that modulated biological pathways in 2DD cells outperformed the other fractions/extracts and had a phenolic molarity of 115.0 μM. Cell treatments included each individual phenolic (4; CA, CY, K3G, and GA); binary combinations (6; CA/CY, CA/K3G, CA/GA, CY/K3G, CY/GA, and K3G/GA); a quaternary combination (1; All 4); and a DMSO vehicle control (1; DMSO). All treatments were performed for 72 h in duplicate or triplicate for each of the assays. As DMSO was not employed to increase cell membrane permeability but rather to afford phenolic solubilization, its concentration was maintained at <0.5% (*v*:*v*) in the media. 

Hydrogen peroxide (H_2_O_2_) was employed to promote intracellular oxidative stress. To do this, the cells were treated with the phenolics as described above for 71.5 h followed by 2 × 10 s washes with unsupplemented DMEM to remove any extracellular phenolics and a 30 min 150.0 μM H_2_O_2_ diluted in unsupplemented DMEM at 37 °C.

### 2.3. Population Doubling Time and Cell Viability 

Cells were seeded at a density of 3000/cm^2^ with 2.0 mL of DMEM in 6-well plates (Thermo Fisher, Rochester, NY, USA) and incubated for 24 h at 37 °C to afford sufficient time for adherence to the plate surface. Following incubation, media was aspirated and replaced with treatment-supplemented media, and cells were incubated for 72 h at 37 °C. After this treatment period, the media was aspirated and 1.0 mL of TrypLE Express was added to each well to dissociate the cells from the plate. The plates were incubated at 37 °C for 5 min or until complete dissociation was visually observed. Following dissociation, the cell suspensions were collected separately from each well and placed in 15 mL falcon tubes. Wells were washed with 1.0 mL of media and this suspension was added to the appropriate falcon tube so as to ensure that all cells (living and dead) were collected. Samples were then centrifuged for 4 min at 174 rcf (Centrifuge 5804 Eppendorf, Hamburg, Germany) and the supernatant was aspirated. The pellet was resuspended in 1.0 mL of fresh media and 10 µL aliquots from each tube were transferred onto clean parafilm. An equal volume of trypan blue dye (10 µL; Gibco, Catalog #: 15250, Grand Island, NE, USA) was then added to each aliquot, and this mixture was incubated at RT for 3 min. Following incubation, two 10 µL aliquots of each sample were individually analyzed for cell counts and cell viability with a hemocytometer (Countess II FL, Thermo Fisher Scientific). All treatments were performed with a minimum of three biological replicates (*n* = 3).

Population doubling time was calculated with the following formula:

$$PDT\left(h\right)=incubation\;time\left(h\right)\;\times \frac{\log\left(2\right)}{\log\left(\frac{final\;cell\;count}{initial\;cell\;count}\right)}$$

Percentage cell viability was calculated with the following formula:

$$\%\;viability=100\%\times \frac{total\;counted\;cells-nonviable\;cells}{total\;counted\;cells}$$

### 2.4. RNA Extraction 

Cells were plated in T25 plates (VWR, Visalia, USA; Cat #: 353014; 25 cm^2^) and treated as previously described (Phenolic Treatments). Following the 72 h treatment period, 1 mL of TRIzol reagent was added to the plate and cells were scraped from the surface with a disposable cell lifter (Fisher; Cat #: 08-100-240). Samples were stored at −80 ± 4 °C until time of extraction.

The following steps were all conducted with samples placed on ice, and all centrifugations at 4 ± 2 °C. Samples stored in Trizol at −80 ± 4 °C were thawed on ice. Initially, 200 µL of chloroform was added and samples were vortexed (Baxter Diagnostics Inc., Deerfield, IL, USA; Cat #: S8223-1) at speed 9 for 10–20 s. Samples were then incubated for 5 min followed by centrifugation (Eppendorf Centrifuge 5424 R; Mississauga, ON, Canada) at 21,130 rcf for 10 min. The aqueous phase (top layer; ∼300–500 μL) was collected and transferred into a new 1.5 mL centrifuge tube. To precipitate RNA, 3M sodium acetate (pH 5.2) was added at 1/10 of the volume of the aqueous phase (~60 μL) and vortexed. Following this, an equal volume to the aqueous phase of isopropanol (~300–500 μL) was added and samples were vortexed. Samples were then left static on ice for 30 min followed by centrifugation at 21,130 rcf for 10 min to pellet RNA. The supernatant was removed and 1.0 mL of 70% ethanol (*v*:*v*; 4 ± 2 °C) was added to wash the pellet followed by centrifugation at 21,130 rcf for 10 min. The supernatant was removed, and the RNA pellet was air-dried for 10 min. 

A Dnase treatment was performed by resuspending the air-dried RNA pellet in 88 µL of nuclease free water, followed by the addition of 1 µL RNaseOUT^TM^, 10 µL of 10× DNase I reaction buffer, and 1 µL DNase I. Samples were then vortexed and incubated for 20 min at 37 °C. Following incubation, 100 µL of acid phenol/chloroform (5:1 (*v*:*v*); pH 4.5) was added, samples were vortexed and centrifuged at 21,130 rcf for 15 min. The aqueous phase (100 µL) was collected and transferred into a new 1.5 mL centrifuge tube and 1/10 the volume (~10 μL) of the aqueous phase of 3M sodium acetate (pH 5.2) was added to the collected aqueous phase followed by vortexing. Absolute ethanol (4 ± 2 °C) was then added to each sample at a volume of 3× the volume of the aqueous phase followed by vortexing and precipitation for 30 min (4 ± 2 °C). Following the second precipitation, the samples were centrifuged at 21,130 rcf for 30 min. The supernatant was removed, and the RNA pellet was air-dried for 10 min. The air-dried pellet was resuspended in 30.5 µL of nuclease free water and 1 µL of RNaseOUT^TM^ was added. All samples were stored at −80 ± 4 °C until cDNA synthesis. To confirm if RNA extraction was successful, 5 µL of each extracted sample was run at 90 V on a 1% agarose gel. Samples that exhibited clear 18S and 28S bands, indicating successful RNA extraction, were used for cDNA synthesis. Sample RNA concentration was quantified employing UV spectroscopy at 260 nm using a NanoDropTM 2000 Spectrophotometer (Thermo Fisher, Wilmington, DE, USA).

### 2.5. Complementary DNA (cDNA) Synthesis and Reverse Transcriptase Quantitative Polymerase Chain Reaction (RT-qPCR)

RNA samples were thawed on ice followed by the addition of 1 µL of random primers (50 ng/µL) and 1 µL of dNTPs (10 mM) and brought to a final volume of 13 µL with nuclease free water. The solution was mixed and heated for 5 min in a Thermomixer (Fisher Scientific) at 65 ± 2 °C to denature the RNA secondary structure. Samples were then placed on ice and left stagnant for 1 min. The following reagents were then added: 4 µL 5× First Strand Buffer, 1 µL of 0.1 mM DTT, 1 µL of RNase out, and 1 µL of Superscript III reverse transcriptase. The solution was incubated at RT for 5 min and then in the Thermomixer for 60 min at 50 °C to afford cDNA synthesis. The reaction was inactivated by heating to 70 °C for 15 min. Finally, the samples were brought to a volume of 200 µL with nuclease free water and stored at −20 ± 2 °C until analysis.

Following cDNA synthesis, RT-qPCR was performed by combining 5 μL PerfeCTa^®^ SYBR^®^ Green Supermix, 2 μL cDNA template, 1 μL nuclease free water, and 1 μL primer mix (3 μM forward and reverse). Non-template controls were run for each primer pair (Table 1) and three technical replicates of three biological replicates (*n* = 3) were performed per treatment. The reactions were performed employing a Rotor-Gene^®^ qPCR machine (Quiagen, Germantown, MD, USA). Melt curves were produced and analyzed to ensure that only a single product was synthesized in each reaction. Results were quantified employing the ΔΔCt method to calculate fold changes based on the average of three normalizing genes (EFEMP2, FAU, and FKBP10). Primer pairs for each reaction were designed employing the Primer 3 software (version 0.4.0). Fold changes were calculated as follows:

ΔCt treatment = Ct target gene in treatment − Ct normalizing genes in treatment ΔCt = Ct target gene in control − Ct normalizing genes in control ΔΔCt = ΔCt treatment − ΔCt control Fold change = 2^−ΔΔCt^

Three housekeeping genes were used in this research to ensure that if there is an anomalous treatment impact on one of these genes, this does not distort the data. Therefore, the use of three housekeeping genes increases confidence that an observed change is biologically relevant. These were selected on previous research [29,30] utilizing these cells. For the fold change calculations, each sample had the absolute value fold change (|fold change|) calculated for each housekeeping gene individually; then the three values obtained from each housekeeping gene were averaged. Following this, the average for each biological replicate was calculated to generate the final fold change value. 

### 2.6. Protein Extraction, Quantification, and Western Blotting 

Cells were grown and treated in 6-well plates. Following the 72 h treatment period, cell media was aspirated and the cells were washed (2 × 10 s) with 2 mL of unsupplemented DMEM. For the hydrogen peroxide (H_2_O_2_)-treated cells, 2 mL of unsupplemented DMEM containing 150.0 µM H_2_O_2_ was added to each well, followed by incubation for 30 min at 37 °C. Following this, the DMEM was aspirated and 100 µL of Laemmli buffer (62.5 mM Tris-HCl pH 6.8, 2% sodium dodecyl sulfate (SDS; *w*:*v*), 10% glycerol (*v*:*v*), and 5% 2-mercaptoethanol (*v*:*v*)) containing protease inhibitor cocktail 2 and phosphatase inhibitor cocktail 2 each at a ratio of 100:1 (*v*:*v*), was added to each well. Cells were scraped from the plate surface and collected into a 1.5 mL centrifuge tube followed by 20–30 s vortexing (S/P^®^ Vortex Mixer, Baxter). The lysates were then centrifuged (21,000 rcf, 4 °C) for 5 min to preclear insoluble cell components and the supernatant was collected into a new 1.5 mL centrifuge tube. Protein lysates were stored at −20 ± 3 °C until time of use. Protein sample concentrations were quantified employing a UV NanoDrop^TM^ 2000 Spectrophotometer (Thermo Fisher, Wilmington, DE, USA) at 280 nm; the blank was Laemmli buffer. Lysates were brought to equal concentrations with Laemmli buffer and 5× SDS-PAGE loading buffer (50 mM Tris-HCl p 6.8, 6% glycerol (*v*:*v*), 0.0005% Bromophenol blue (*v*:*v*), 4% 2-mercaptoethanol (*v*:*v*, and 2% SDS (*w*:*v*)). Lysates were then denatured in a 95 °C Dry Bath Incubator (Thermo Fisher Scientific) for 5 min. Following denaturation, samples were spun down in a Galaxy MiniStar centrifuge (VWR) to collect any liquid that may have condensed on the sample tube lid during the denaturation step.

Samples (10 μg of protein per well) were loaded into an 8% polyacrylamide gel with a 5% stacking gel and proteins were separated by running at 110 V for 90 min (PAGE; Bio-Rad, Hercules, CA, USA) while submerged in ~1 L of 1× SDS-PAGE running buffer (25 mM Tris base, 192 mM glycine, and 0.1% SDS). To ensure equal protein loading for each protein lysate, a Coomassie blue assay was run. Following separation as previously described, the gel was incubated in 80–100 mL of a Coomassie fixative solution (25% isopropyl alcohol (*v*:*v*) and 10% glacial acetic acid (*v*:*v*)) and placed under agitation for 1–2 h at RT. Coomassie fixative solution was removed and replaced with 80–100 mL of Coomassie Stain solution (10% glacial acetic acid (*v*:*v*) and 0.006% Coomassie Blue R-250 (*ไ*:*v*)) under agitation for 1–2 h at RT and left stagnant for ~15 h at 4 ± 2 °C. The Coomassie stain solution was removed and replaced with 80–100 mL of Coomassie Destain (10% glacial acetic acid (*v*:*v*)) and placed under agitation for 1–2 h at RT until imaged. 

Following separation, the protein lysates were run as previously described and transferred onto a nitrocellulose membrane (Bio-Rad Laboratories, Hercules, CA, USA, Catalog #: 1620112). Transfer (i.e., blotting) was performed employing a wet transfer apparatus that was submerged in ∼1 L of wet transfer buffer (25 mM tris base, 192 mM glycine, 20% methanol (*v*:*v*), and 0.04% SDS) and run at 0.23 Amps under agitation (stir bar and plate) at RT for 90 min. Following transfer, the membranes were blocked in 5% skimmed-milk powder (SKM) in phosphate-buffered saline with Tween^TM^ 20 (PBST; pH 7.4; 137 mM sodium chloride, 2.7 mM potassium chloride, 8.1 mM disodium hydrogen phosphate, 1.47 mM potassium dihydrogen orthophosphate, and 0.05% Tween^TM^ 20 (*v*:*v*)) (SKM-PBST) rotating at RT for 1 h. The membrane was then transferred into a 50 mL flacon tube containing 5 mL of 2.5% SKM-PBST and the mouse anti-SIRT1 primary antibody (Abcam, Cambridge, MA, USA, Catalog #: ab110304) at 1:1000 dilution. The membrane was left to incubate with the primary antibody for ~15 h rotating at 4 ± 2 °C.

Membranes were imaged employing the iBright 750 (Thermo Fisher Scientific) and enhanced chemiluminescent reagents (ECL; 0.2 mM *p*-coumaric acid, 1.25 mM luminol, 100 mM Tris-HCl pH 8.5, and 0.1% hydrogen peroxide (*v*:*v*)). Protein bands were quantified employing ImageJ software (version Java 1.8.0-172 (64-bit); https://imagej.nih.gov/ij/ accessed on 1 September 2021) densitometry measurements. A Coomassie load control was employed to ensure equal loading of proteins and bands were first normalized to the load and then to the control sample (DMSO). Three biological replicates for each cell line were performed (*n* = 3).

### 2.7. Immunofluorescence 

Cells were grown on glass coverslips (Fisher; Cat #: 12548B; size 22 × 22-1) in 6-well plates and treated as previously described (Phenolic Treatments). Following the treatment period, cells on coverslips were fixed with 2.0 mL of 3.7% formaldehyde in PBS (*v*:*v*) for 10 min at room temperature (20 ± 3 °C; RT). Formaldehyde was removed and the cells were washed (2 × 5 min) with PBS at RT. Cells were dehydrated in the following series (1 × 5 min) H_2_O, 50% ethanol, 70% ethanol, 90% ethanol and absolute ethanol; cells were stored in absolute ethanol at −20 ± 3 °C until time of use. Cells were rehydrated in the following series (1 × 5 min) 90% ethanol, 70% ethanol, 50% ethanol, H_2_O, and PBS. Cells were then permeabilized with 0.5% Triton X-100 for 10 min at RT followed by (1 × 5 min) washing with PBS and placed in 0.05% Triton X-100.

Excluding 07124B H_2_O_2_, cells were blocked with 40 µL per coverslip of 1% bovine serum albumin in PBS (*w*:*v*; 1% BSA) for 30 min in a humidity chamber. The humidity chamber consisted of a glass plate, 0.05% Triton X-100, parafilm, a 6-well plate lid, and a box. Cells were removed from the blocking humidity chamber, with excess liquid dabbed employing a kimwipe, and placed in a new humidity chamber at RT with 25 µL per coverslip of primary antibody at a predetermined dilution (1:50–1:200; dependent on antibody strength) in 1% BSA for 1 h followed by washing (1 × 5 min) in 0.05% Triton X-100. Cells were then incubated with the secondary antibody at 1:200 dilutions following the same protocol. Secondary antibodies conjugated to fluorophores included either goat anti-mouse 488 (excitation/emission max; 493/518 nm) or Cy3 (excitation/emission max; 555/596 nm) or donkey anti-rabbit 488 or Cy3, depending upon the species of the primary antibody used.

Cell nuclei were counter-stained with 15 µL VECTASHIELD Mounting Medium with DAPI (excitation/emission max; 359/461 nm, Cat #: H-1200), mounted onto glass slides (Fisher Scientific; Cat #: 12-550-403; size 25 × 75 × 1 mm) and sealed with nail varnish, stored at 4 ± 2 °C until imaging. Imaging was conducted by fluorescence microscopy at 40× and 100× magnification with constant exposure times (Olympus X51, Tokyo, Japan). For visualization by fluorescence microscopy, white light (λ = 400–700 nm) was passed through a filter, which permitted wavelengths of blue (λ = 461) or red (λ = 596) light through the sample to excite the fluorophore for visualization (i.e., signal). A minimum of 35 cells per sample replicate were imaged and a minimum of two biological replicates (*n* = 2) were conducted for each treatment condition.

### 2.8. SIRT1 Activity Assay

Cells were grown in T25 plates (Corning; Cat #: 353014; 25 cm^2^) and treated as previously described (Phenolic Treatments). Following the 72 h treatment period, cells were dissociated from the plate with 2.0 mL of TrypLE Express and incubated at 37 °C until complete dissociation was observed. The TrypLE Express cell suspension was collected and transferred into appropriately labeled 15 mL Falcon tubes. Plates were washed with 3.0 mL of DMEM to collect any remaining cells with this wash suspension added to the appropriate tube. Tubes were centrifuged at 174 rcf for 4 min to pellet cells. Following centrifugation, the supernatant was aspirated and the cell pellet was resuspended in 50 μL of non-denaturing lysis buffer (50 mM Hepes KOH (pH 7.5), 420 mM NaCl, 0.5 mM EDTA disodium salt, 0.1 mM egtazic acid, and 10% glycerol (*v*:*v*)). This volume was transferred into an appropriately labeled 1.5 mL centrifuge tube and the 15 mL tube was washed with another 50 μL of non-denaturing lysis buffer with this solution transferred into the appropriate 1.5 mL centrifuge tube. Sample protein content was quantified employing UV spectroscopy at 280 nm using a NanoDropTM 2000 Spectrophotometer (Thermo Fisher, Wilmington, DE, USA). Samples were brought to equal concentration with non-denaturing lysis buffer followed by vortexing, and stored at −80 ± 5 °C until analysis.

SIRT1 activity levels were measured fluorometrically employing an assay kit (Abcam, Cat #: ab156065). Reactions were prepared in a 96-well black microtiter plate (Greiner Bio-one, Cat #: 675076) at 4 ± 2 °C. The assay solution was prepared by adding 5 μL of H_2_O, 5 μL Sirtuin1 assay buffer, 5 μL Trichostatin A (10 μM in DMSO), and 5 μL 2 mM nicotinamide dinucleotide (NAD), and this solution was mixed well. A total of 5 μL of the fluoro-substrate peptide and developer was added and mixed followed by the addition of 20 μL of treated lysates in non-denaturing lysis buffer. Negative controls were run with 20 μL of H_2_O and positive controls were run with 5 μL of recombinant SIRT1. The instrument employed for this assay was the Varioskan Lux by Thermo Scientific (Cat #: VL000D0) with SkanIt RE 6.0 software. Instrument settings were as follows, fluorescence at 350 nm excitation and 460 nm emission was measured and readings were taken every 2 min for 1 h at 37 °C with plate shaking for 5 s every 1 min 50 s. Three technical replicate measurements were taken per two biological replicates (*n* = 2). Final reaction volumes of 50 μL were maintained for all samples. Following 1 h of fluorescence measurements, the final time point fluorescence values were used for analysis. Results were normalized by dividing the treated sample fluorescence by the vehicle control (DMSO) fluorescence and results are presented in arbitrary fluorescence units.

### 2.9. Statistical Analysis 

The statistical analysis of the experimental data was performed using R Studio version 3.2.3 for Windows. A general linear mixed effects model with a random intercept model was employed. Biological replicates were set as the random factor to account for any potential random variables that may cause non-independence between replicates. The library nlme and a lme model were employed. The estimated marginal means (emmeans) was used for post hoc testing and as its pairs function affords multiple pairwise comparisons. 

## 3. Results

### 3.1. Phenolic Treatments Differentially Impact Cell Population Doubling Times Without Increasing Cell Death

For the purpose of this research, we selected four structurally distinct phenolics previously identified by Zehfus and colleagues [20] using mass spectrometry of haskap phenolic fractions/extracts. These compounds (CA, CY, K3G, and GA) are representatives of major phenolic classes/subclasses identified and significantly were also selected based on structural differences, with each compound containing unique features (Figure 1). CA and GA contain one phenolic ring with two hydroxyl groups. They differ in the arrangement of the hydroxyls around the ring with respect to each other but also the position of the carboxylic acid group. Additionally, they differ in the alkyl group length of carboxylic acid. CY and K3G are polyphenols, meaning they each contain two or more phenolic rings, but they differ in the number and locations of hydroxyl groups. K3G contains a β-D-glucosyl group at the C3 position whereas CY contains a C3 hydroxyl. K3G also contains a carbonyl group at C4 that is absent in CY. CA and CY share a catechol group (3,4-dihydroxy) that is absent in K3G and GA.

**Figure 2:**
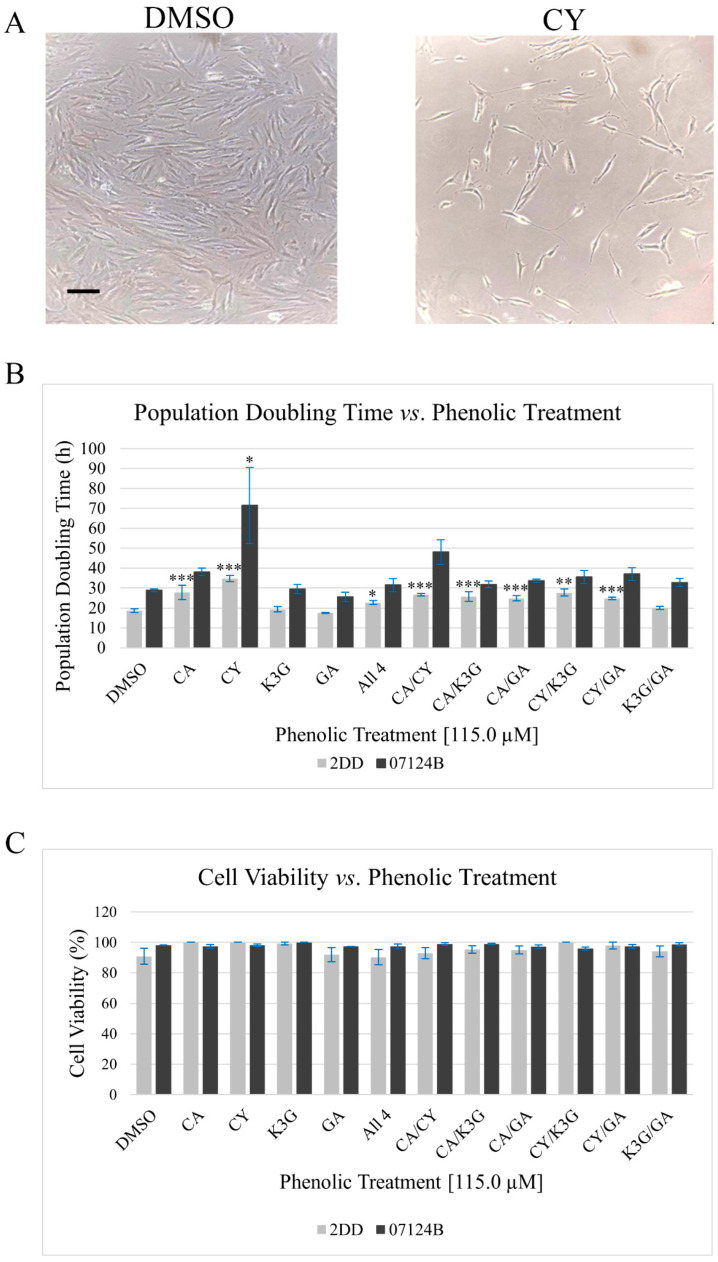
The phenolic treatments have structure-dependent impacts on 2DD and 07124B cell growth behavior. (**A**) Representative images of DMSO and CY-treated 2DD cells following a 72 h treatment period. Scale bar = 100 μm. (**B**) Quantified PDT assay results. Following the treatment period, cells were collected and counted affording the calculation of PDT that was normalized to the vehicle control (DMSO). Error bars represent the standard error of the mean (*n* = 3; *p* = * *p* < 0.1, ** *p* < 0.05, *** *p* < 0.01). (**C**) Cell viability assay results, the phenolic treatments maintained high levels of cell viability indicating that these compounds did not increase 2DD or 07124B cell death. Error bars represent the standard error of the mean (*n* = 3). Treatment abbreviations are as follows: vehicle control (DMSO), caffeic acid (CA), cyanidin (CY), kaempferol-3-*O*-glucoside (K3G), and gentisic acid (GA).

Ref. [20] observed that haskap phenolic extracts and fractions increased normal and immortalized primary human fibroblast PDT. From these observations using complex mixtures of phenolics, the authors hypothesized that the selected phenolics would differentially and synergistically impact cell growth behavior, and it is predicted that the phenolics will increase cell PDT. To provide evidence in support of this hypothesis, we exposed two normal primary human fibroblast lines to CA, CY, K3G, GA or their binary or quaternary combinations for 72 h. We maintained a consistent concentration of phenolics at 115.0 μM as this was the total concentration of the aforementioned 40% ethanol haskap phenolic fraction previously used [20]. For phenolic combinations, equimolar amounts of each (57.5 μM of each phenolic and 28.74 μM for the quaternary) were added to culture media. Following the treatment period, the PDT assay was employed. Representative images and quantified PDT results, comparing the phenolics to the control DMSO, are presented in Figure 2A and Figure 2B, respectively.

Results indicated that the phenolics had structure- and combination-dependent impacts on 2DD and 07124B PDT (*p* < 0.001–1.000) (Supplemental Table S1). In both cell lines, the CY treatment resulted in the largest increase of 16.1 and 42.7 h in PDT for 2DD and 07124B, respectively. The individual phenolic treatments demonstrated the following increased PDT trend CY > CA > K3G/GA, with K3G and GA having no observable impact. These observations were consistent in both cell lines. Our results demonstrated that there were no synergistic effects, with no combination of phenolics outperforming their respective individuals. In 2DD cells, CY/K3G (9.1 h increase) had the largest impact followed closely by CA/CY (8.1 h increase) and the phenolic combinations demonstrated the following increased PDT trend: CY/K3G > CA/CY > CA/K3G > CA/GA > CY/GA > All 4 > K3G/GA. In 07124B cells, CA/CY was the combinatory treatment with the largest impact (19.3 h increase) on PDT, although this value did not reach the threshold of significance (*p* = 0.317). In 07124B cells, the phenolic combinations demonstrated the following increased PDT trend: CA/CY > CY/GA > CY/K3G > CA/GA > K3G/GA > CA/K3G > All 4. 

There are two mechanisms by which cell PDT can be increased: (1) increased cell death; or (2) slowed cell growth. To investigate which mechanism was responsible for the observed increased PDT and to determine if the phenolics were cytotoxic at the experimental concentrations, Trypan Blue was employed to ascertain if the phenolics increased rates of cell death. Results (Figure 2C) indicated high levels of cell viability (90.3–100%) in response to all phenolic treatments in both cell lines, leading the authors to suggest that the observed increased PDT was due to the phenolic treatments slowing cell growth and that these impacts were dependent on phenolic structure. As there is an established connection between lifespan and SIRT1, these results justify further investigations into the phenolic treatments impact(s) on SIRT1.

### 3.2. Phenolic Treatments Had No Significant Impact(s) on SIRT1 Transcript Abundance but Have Structure, Combination, and Cell Line-Dependent Impacts on *SIRT1* Protein Abundance

**Figure 3:**
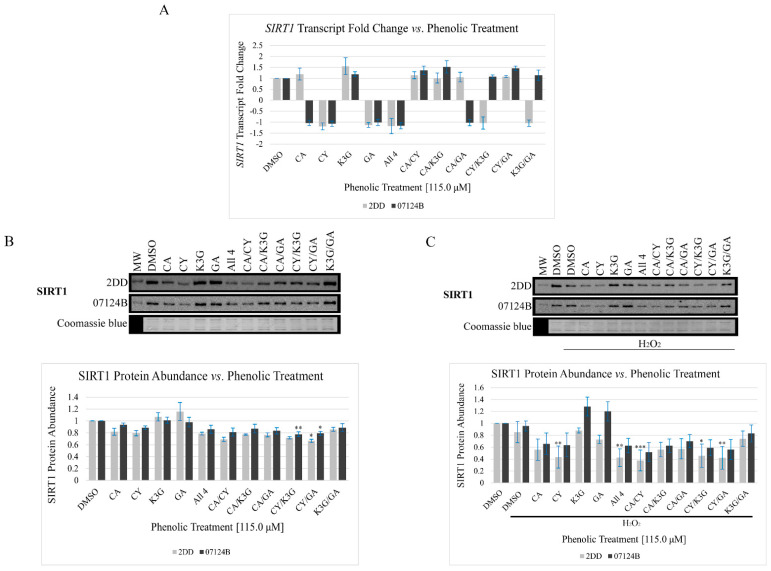
The phenolic treatments had no significant impact on *SIRT1* transcript abundance but had structure-, interaction-, and/or concentration-dependent impacts on SIRT1 protein abundance. (**A**) Cells were treated followed by RNA extraction, cDNA synthesis, and RT-qPCR, a fold change of ±2 was considered significant. (**B**) Impacts of the phenolic treatments on SIRT1 protein abundance. Cells were treated followed by whole cell lysis, harvesting, and Western blot (*n* = 3), representative Western blot and quantified densitometry results are presented. Coomassie blue gels were used as a load control. (**C**) Impacts of the phenolic treatments on SIRT1 protein abundance in response to H_2_O_2_ (*n* = 3; *p* = * *p* < 0.1, ** *p* < 0.05, *** *p* < 0.01). Abbreviations are as follows: vehicle control (DMSO), caffeic acid (CA), cyanidin (CY), kaempferol-3-*O*-glucoside (K3G), gentisic acid (GA), molecular weight (MW), and hydrogen peroxide (H_2_O_2_).

There are numerous mechanisms by which phenolics can regulate SIRT1 transcript and protein abundance. Zehfus and colleagues [20] observed that haskap berry phenolic extracts and fractions had different impacts on SIRT1 protein abundance. Therefore, the authors hypothesized that the selected phenolic treatments would differentially and synergistically impact *SIRT1* transcript abundance, with an increase in transcript predicted. To test this, 2DD and 07124B cells were treated with the selected phenolics for 72 h followed by harvesting, RNA extraction, cDNA synthesis, and RT-qPCR. RT-qPCR results (Figure 3A, Supplemental Table S2) indicated that the phenolic treatments had no significant impact on *SIRT1* transcript abundance in 2DD or 07124B cells. Although slight variations in transcript abundance were observed, a significance threshold of ±2-fold change was selected for this work, and no phenolic treatment surpassed this threshold.

Changes, or the lack of changes, in transcript abundance are not always direct readouts of alterations in protein abundance [31]. Ref. [20] reported that haskap berry phenolic extracts/fractions modestly increased SIRT1 protein abundance (<3-fold increases). As such, the selected phenolic treatments’ impact on SIRT1 protein abundance were investigated. To do this, 2DD and 07124B cells were treated with the selected phenolics for 72 h followed by harvesting of whole cell lysates, protein quantification, and Western blot (Figure 3B). Results (Figure 3B, Supplemental Table S3, Supplemental Figure S1) indicated that the phenolic treatments had structure-dependent impacts on SIRT1 protein abundance and, surprisingly, decreases were observed. The phenolics demonstrated similar impacts on SIRT1 protein abundance in both cell lines. Nine of the eleven phenolic treatments decreased SIRT1 protein abundance, although in 2DD cells only one of these treatments (CY/GA) reached the threshold of significance (*p* ≤ 0.10) and only two treatments (CY/K3G and CY/GA) in 07124B cells. In 2DD and 07124B cells, the individual phenolics demonstrated the following decreased SIRT1 trend: CY > CA > K3G/GA. In 2DD cells, the CY/GA treatment had the largest impact on SIRT1 protein abundance with a 34% decrease (*p* < 0.10). The combinatory treatments in 2DD cells demonstrated the following decreased SIRT1 trend: CY/GA > CA/CY > All 4 > CY/K3G > CA/GA > CA/K3G > K3G/GA. 

In 07124B cells, CY/K3G and CY/GA had the largest impacts on SIRT1 protein abundance. CY/K3G had the largest impact on SIRT1 levels with a 22% decrease (*p* > 0.05) and the combinatory treatments demonstrated the following decreased SIRT1 trend: CY/K3G > CY/GA > CA/CY > CA/GA > All 4 > CA/K3G > K3G/GA. In both cell lines, generally, any phenolic treatment that contained CY had a larger impact than the treatments without CY. Moreover, CY/GA was the only treatment that had significant impacts on SIRT1 protein abundance in both cell lines.

SIRT1 has established regulatory roles in the cellular response to oxidative stress [32,33]. Therefore, to further the investigation into the phenolic treatment impacts on SIRT1 protein abundance in 2DD and 07124B cells, the cells were treated with the phenolics for 71.5 h followed by a 30 min 150 µM H_2_O_2_ treatment, cell lysate harvesting, and Western blot (Figure 3C). Results (Figure 3C, Supplemental Table S3) indicated that, when exposed to oxidative stress (H_2_O_2_), the phenolics demonstrated structure and combination-dependent impacts on decreasing SIRT1 protein abundance. In 2DD cells, all eleven of the phenolic treatments decreased SIRT1 protein abundance, although only five of these treatments reached the threshold of significance (*p* < 0.1). The largest impact was observed with CA/CY, which demonstrated a 63% decrease in SIRT1 levels (*p* < 0.01); this treatment had a similar impact to CY individually. Of note, in 2DD cells, all treatments containing CY resulted in a statistically significant decrease in SIRT1 protein abundance. The individual phenolics in 2DD cells demonstrated the following decreased SIRT1 trend: CY > CA > GA > K3G. In 2DD cells, the combination treatments demonstrated the following decreased SIRT1 trend: CA/CY > CY/GA > All 4 > CY/K3G > CA/K3G > CA/GA > K3G/GA, although the decreases observed with CA/K3G, CA/GA, and K3G/GA were not statistically significant. In 07124B cells, nine of the eleven phenolic treatments decreased SIRT1 protein abundance and two (K3G and GA) of the eleven phenolic treatments increased SIRT1 levels; however none of these observed impacts surpassed the threshold of significance (*p* < 0.1). Interestingly, in the 07124B cells, K3G and GA increased SIRT1 levels; however, when they were combined (K3G/GA), a decrease was observed. Of note, in both cell lines, H_2_O_2_ treatment of the DMSO control had no observable impact on SIRT1 protein abundance.

### 3.3. Phenolic Treatments Do Not Impact *SIRT1* Localization, but Had Structure, Combination, and Cell Line-Dependent Impacts on *SIRT1* Activity Level

SIRT1 function is, in part, regulated by its sub-cellular localization. Therefore, we hypothesized that the selected phenolic treatments would differentially and synergistically impact SIRT1 cellular localization under baseline and oxidative stress conditions. To test this, 2DD and 07124B cells were grown on coverslips, treated with the phenolics for 72 h, fixed, and immunolabeled as per the immunofluorescence assay. 

**Figure 4:**
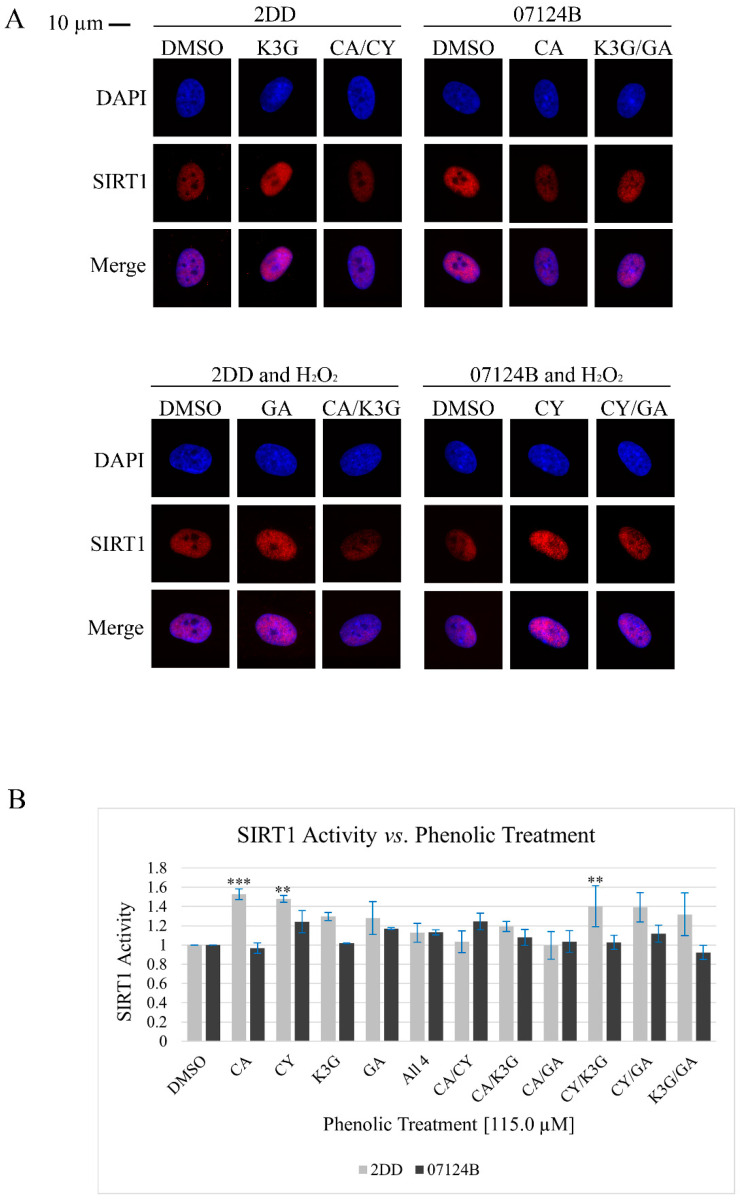
The phenolic treatments had no observable impact on SIRT1 localization but had structure and cell line-dependent impacts on SIRT1 activity level. (**A**) Representative immunofluorescence images. Chromatin was counter-stained with DAPI and is represented by the blue color, SIRT1 is represented by the red color. Greyscale images were false colored in Photoshop and all treatments were imaged with consistent exposure times and treated equally in Photoshop to avoid artifact creation. The DAPI (blue) and SIRT1 (red) images were overlayed to create the Merge image, where a purple color suggests co-localization of chromatin and SIRT1. A total of 30–50 cells were imaged per treatment replicate (*n* = 2). (**B**) SIRT1 activity assay results. Cells were treated with the selected phenolics prior to harvesting in non-denaturing lysis buffer. Error bars represent the standard error of the mean and fluorescence values were normalized to DMSO (*n* = 2; *p* = ** *p* < 0.05, *** *p* < 0.01). Treatment abbreviations are as follows: vehicle control (DMSO), caffeic acid (CA), cyanidin (CY), kaempferol-3-*O*-glucoside (K3G), gentisic acid (GA), and hydrogen peroxide (H_2_O_2_).

Immunofluorescence results (Figure 4A) in both 2DD and 07124B cells under both conditions indicated that SIRT1 was primarily localized to the nucleus and more specifically the nucleoplasm. SIRT1 was also identified, to a small degree, in the cytoplasm; however, as the exposure times required for imaging to visualize cytoplasmic SIRT1 signal caused the nuclear SIRT1 to be overexposed. Results indicated that none of the phenolic treatments had an observable impact on SIRT1 localization with or without oxidative stress.

The level of SIRT1 deacetylase activity, which is independent of localization, may be impacted by the phenolic treatments. Zehfus and colleagues [20] reported that haskap phenolic extracts/fractions had indirect impacts on SIRT1 activity in 2DD and Nb1hTERT cells. Therefore, the authors predicted that the selected phenolics would differentially and synergistically increase SIRT1 activity in 2DD and 07124B cells. To test this, 2DD and 07124B cells were treated with the selected phenolics for 72 h followed by harvesting into a non-denaturing lysis buffer and a SIRT1 activity assay. This assay, in short, uses a fluorescent probe, which upon deacetylation will provide information on SIRT1 activity level. As SIRT1 is not the only deacetylase present in cells, the selective inhibitor trichostatin A was employed as SIRT1 is resistant to its inhibition, but other deacetylases are not. As such, the selected phenolic treatment impacts on SIRT1 activity were determined with this SIRT1 activity assay.

SIRT1 activity assay results (Figure 4B, Supplemental Table S4) indicated that the phenolic treatments had structure, combination, and cell line-dependent impacts on SIRT1 activity. In 2DD cells, eight of the eleven treatments increased SIRT1 activity, although only three of these treatments (CA, CY, and CY/K3G) surpassed the threshold of significance (*p* ≤ 0.1). Three treatments (All 4, CA/CY, and CA/GA) had no observable impact. In 2DD cells, the individual phenolics demonstrated the following increased SIRT1 trend: CA > CY > K3G/GA and the phenolic combinations increased SIRT1 activity in the following trend: CY/K3G > CY/GA > GA/K3G > CA/K3G. No combination increased SIRT1 activity greater than CA or CY individually and no synergy was observed between the selected phenolics under these experimental conditions. 

In 07124B cells, three of the eleven phenolic treatments (CY, GA, and CA/CY) increased SIRT1 activity; however none of these observed impacts surpassed statistical significance (*p* < 0.1). The remaining eight treatments had no observable impact. CY resulted in the largest increase in SIRT1 activity. CA/CY was the only combination that increased SIRT1 activity; however, as this impact was less than that of CA and CY individually, no synergy was observed in the 07124B cells amongst these phenolics with respect to this function. Overall, the impact of the phenolic treatments on SIRT1 activity was smaller in 07124B cells compared to 2DD cells.

## 4. Discussion

We had previously determined that phenolic extracts from haskap berries had impacts on readouts of cellular stress that was dependent on the presence of SIRT1, and that these complex phenolic mixtures increased SIRT1 activity in normal and immortalized human male fibroblasts [20]. To further investigate the impact of phenolics on SIRT1, the aim of this work was to determine (1) if individual phenolics and their associated structures or their combinations, through synergy, were required to impact SIRT1; and (2) identify if the phenolics or their combinations impact SIRT1 transcript and protein abundance, localization and/or activity. Four structurally different phenolics that have been identified in haskaps were selected (Figure 1). These phenolics were supplemented to normal primary human fibroblasts (2DD [XY] and 07124B [XX]) individually, to determine their functions, and in equimolar combinations to determine if synergy occurs with respect to these functions. 

It was observed that CA, CY, and their respective combinations increased PDT whereas K3G, GA, and K3G/GA had no observable impact on cell growth in both cell lines. The observed differences are hypothesized to be due to phenolic structure, with the phenolics that contain a catechol group (*ortho*-dihydroxy; CA and CY (B-ring)) outperforming those without (K3G and GA). None of the phenolics reduced 2DD or 07124B cell viability, therefore the authors suggest that the phenolics included in this work are not cytotoxic and that they impact pathways that regulate growth. The phenolic combination results indicated that phenolic impacts on 2DD and 07124B were combination-dependent. Alternatively, these results could be a phenolic concentration effect as a linear relationship between PDT and phenolic concentration was not established in this work. However, published data does support the hypothesis that the relationship between cellular growth, viability, and phenolic treatments are linear in nature [30,34]. Previous reports have demonstrated that an increase in population doubling time (PDT), described as a slowing of cell cycle progression without increased cell death leading to an increased time the cells are viable in culture, is associated with SIRT1 and longevity via changes in gene expression profiles that promote maintenance and repair processes over proliferation [20,35,36].

The phenolic treatments did not have a significant impact on *SIRT1* transcript abundance, indicating that transcription of *SIRT1* is not a mechanism by which the selected phenolics impact 2DD and 07124B cells. The phenolics had differing impacts on SIRT1 protein abundance, with the catechol-containing phenolics having a larger impact than those without in both cell lines. The same impacts on SIRT1 protein abundance were observed in the presence and absence of oxidative stress in both cell lines. These results further support the hypothesis that the catechol structure is of significance for fibroblasts. With respect to the phenolic combinations, CY/GA decreased SIRT1 protein abundance more than CY and GA individually in both cell lines under base line conditions. This indicates a combination effect and potential synergy between these phenolics with respect to this function. Although an increase in SIRT1 is associated with longevity, therefore this decrease could be viewed as antagonism; nevertheless this observation is likely due to a combination effect. An additional combination effect was observed in the 07124B cells between CY/K3G. 

SIRT1 has enzymatic targets in both the cytoplasm (e.g., transcription factors such as NRF2 and NF-κB) and the nucleus (e.g., histone and non-histone proteins) and, therefore, SIRT1 can be regulated by its sub-cellular localization [37,38]. Immunofluorescence results indicated that SIRT1 was primarily nuclear and that the phenolics had no observable impact on localization. The identification of nuclear SIRT1 is consistent with the literature stating that in normal cells, SIRT1 is localized to the nucleus [39]. Results presented in this work indicated that the only structural feature shared by these phenolics, the phenolic ring, may not cause SIRT1 to change localization and does not support the hypothesis that the catechol structure is significant to fibroblasts. Alternately, the selected phenolics may have lacked the structural features required for this function. Although, immunofluorescence results indicated differences in the signal intensity between the treatments, representing changes in the level of SIRT1, which was consistent with Western blot results (Figure 3).

Interestingly, the phenolics had different impacts on SIRT1 activity levels. The 2DD cells were more responsive than the 07124B cells; this indicates that there may be sex- and/or individual-dependent responses to phenolics. The observed lack of impacts on SIRT1 activity and decreased protein abundance may be due to the treatment time of the experiments (72 h). This may be too long to observe the height of the potential impacts and the cells may be returning to baseline behavior following the 72 h. SIRT1 activity assay results, excluding CA in 07124B cells, are consistent with the Western blot results in which the catechol-containing phenolics had a larger impact than those without a catechol group. Moreover, no synergy was observed with respect to SIRT1 function but, in 2DD cells, the combination CA/CY had no observable impact on SIRT1 activity despite CA and CY individually having the largest impact. This, in conjunction with combination effects observed in Western blot, leads the authors to suggest that not only can phenolic combinations exhibit synergy, but they may also exhibit antagony. Defining the observed impacts on SIRT1 as synergistic or antagonistic is difficult as the phenolics may be decreasing cell stress, which results in a reduced need for SIRT1 protein and its associated activity levels. However, this would ultimately benefit the cells and, therefore, may be considered a synergistic combination effect. Moreover, although only three of the phenolic treatments increased SIRT1 activity (2DD cells) to a level that surpassed the selected threshold for statistical significance (*p* < 0.1), statistical significance does not directly equate to biological significance and the observed increases in SIRT1 activity, although small, may be physiologically relevant [40,41].

The concept of phenolic combinations exhibiting synergy and antagony in biological systems, and extending this possibility with other classes of compounds, is of increasing scientific interest; however, the details of the mechanisms governing these interactions require further study. Proposed mechanisms for phenolic synergy and antagony include, but are not limited to [15,42] (1) phenolic interactions (e.g., electrostatic, hydrogen bonding, π-cloud overlap, ect.) forming higher order structures that have increased or decreased activity; (2) phenolic structure regeneration where the stronger compound regenerates the weaker and vice versa; (3) solubility differences; and (4) in complex interconnected systems (e.g., cells) the structurally different compounds may impact different pathways resulting in altered effects compared to the individuals. Any of the aforementioned mechanisms, in addition to mechanisms that have yet to be defined, may be responsible for the combination effects observed in the present work. Moreover, to determine if the observed differences between the individual phenolics and the combinations were due to one of the aforementioned mechanisms or concentration effect, a linear relationship between phenolic concentration and function needs to be established. An alternative mechanistic consideration for the observed differences between the phenolics is bioavailability. That is, the phenolics may have different abilities to cross the cell membrane and impact cell processes, although this would also be dependent on structure. With respect to structure, it remains unclear whether the unmodified phenolics or their associated, structurally different, metabolites are responsible for the observed impacts. In addition, phenolic synergy has been demonstrated to be dependent on phenolic ratio, which was also not investigated in the present research [13]. Given our observations and those previously published, we can state that by whatever mechanism, the resulting impact(s) on SIRT1 activity is not the result of phenolics directly interacting with SIRT1 [20].

We demonstrated that the catechol-containing phenolics decreased SIRT1 protein abundance, but increased SIRT1 activity. Therefore, we suggest that there may be a negative feedback mechanism that regulates SIRT1 protein abundance, that is, increased SIRT1 activity causes the cells to reduce SIRT1 protein abundance. Han et al. (2010) [43] reported that SIRT1 binds to its own promoter in Hela cells by chromatin immunoprecipitation and that Hela cells transfected with SIRT1 causes a reduction in SIRT1 transcript abundance.

Cell line-dependent results from this work indicated that the phenolic treatment impacts are not only dependent on phenolic structure, but are also individual- and/or sex-dependent. The differences observed between 2DD and 07124B cells may be explained by inter-individual variation; perhaps these individuals have varied single nucleotide polymorphisms or epigenetic profiles that make them more/less responsive to different phenolic structures. Alternatively, the observed differences may be due to sex differences (varied metabolic requirements/priorities); however, this cannot be concluded from this work. The inclusion of more male and female cells from different individuals would be required to test if these differences are sex related.

Importantly, this work was performed employing in vitro tissue culture of primary normal human fibroblasts and, therefore, provides scientific information on intracellular activities of phenolics. Although organismal/in vivo aging and health is dependent on a continuum of factors (e.g., bioavailability, digestion, pH, metabolites, microbiota, etc.), studying the mechanism at a cellular level is vital to understanding how dietary compounds, such as phenolics, may impact mechanisms governing cell response (aging, disease, and health).

## 5. Conclusions

These data support the hypothesis that haskap berry phenolics have structure and combination-dependent impacts on SIRT1. These impacts may be primarily achieved through impacts on SIRT1 activity and protein abundance. These results, in conjunction with those from [20] support the hypothesis that phenolics impact readouts of cell health independent of antioxidant activity and support further investigations into the effects of phenolics on additional readouts of cell health.

In conclusion, the cellular responses to individual phenolics, or simple phenolic combinations was not as dramatic as complex haskap phenolic extract/fractions previously observed [20]. The current work provides important information for the field in that simple phenolics may not be as effective as nutraceuticals as would be naturally occurring food matrices such as haskap berries. Although there is still considerable debate regarding the bioavailability of dietary compounds, such as phenolics, these observations do provide information on how, mechanistically, phenolics compounds promote cellular longevity. 

## Figures and Tables

**Table 1:** Primer sequences for the primers used in this research, produced with Primer3 software.

**Target Gene**	**Forward Primer Sequence (5′→3′)**	**Reverse Primer Sequence (5′→3′)**
*EFEMP2*	CGGTTCTCAGAGACCTGGATG	GCCCAAACCTGTGTCAACTTC
*FAU*	CGCATGCTTGGAGGTAAAGTC	TTCTCCTGTTTGGCCACCTTA
*FKBP10*	GCCGTGCTAATCTTCAACGTC	GGTGGTCTCATTGCAGGTCTC
*SIRT1*	CCGGATTTGAAGAATGTTGGT	AATCTGCTCCTTTGCCACTCT

## Data Availability

Data are contained within the article and Supplementary Materials.

## Supplementary Materials

The following supporting information can be downloaded at: https://www.mdpi.com/article/10.3390/cells14040295/s1, Figure S1: Representative western blot with 07124B cells illustrating antibody specificity. Per well, 10 μg of protein was loaded, nitrocellulose membrane used, primary and secondary antibodies were diluted 1:1000.; Table S1: Phenolic treatment impacts on 2DD and 07124B cell population doubling times. Table S2: Phenolic treatment impacts on *SIRT1* transcript abundance. Provided are the mean values of three independent biological replicates (*n* = 3) that were normalized to the DMSO control. Table S3: Phenolic treatment impacts on SIRT1 protein abundance. Values provided are the ratios to control, which were calculated by normalizing the quantified value to the load control and then dividing the treatment by the control value. Three independent biological replicates (*n* = 3) were performed per cell line and experimental condition. Table S4: Phenolic treatment impact on SIRT1 activity level normalized to control.

## Abbreviations

The following abbreviations are used in this manuscript:

SIRT1	Sirtuin 1
CA	Caffeic acid
CY	Cyanidin
K3G	Kaempferol-3-*O*-glucoside
GA	Gentisic acid
C3G	Cyanidin-3-*O*-glucoside
DMSO	Dimethyl sulfoxide
