# Supplementary material for: Phenolic Compounds from Haskap Berries Have Structure, Combination, and Cell Line-Dependent Impacts on the Longevity-Associated Deacetylase Sirtuin 1
Source: Cells. 2025 Feb 17;14(4):295. doi: 10.3390/cells14040295 (PMC11854658; doi:10.3390/cells14040295)

Supplemental Table S1. Phenolic treatment impacts on 2DD and 07124B cell population doubling times.

(A) 2DD

Phenolic Treatment	Replicate 1	Replicate 2	Replicate 3	Mean	S.E.M.	*p*-Value
DMSO	18.9	16.6	20.4	18.6	0.9	
CA	19.9	29.3	34.3	27.8	3.4	0.004
CY	34.3	31.5	38.4	34.7	1.6	<0.001
K3G	17.8	22.5	17.6	19.3	1.3	1.000
GA	18.3	17.0	17.6	17.6	0.3	1.000
All 4	25.0	21.9	21.3	22.8	0.9	0.067
CA/CY	27.6	27.6	25.0	26.7	0.7	0.001
CA/K3G	31.5	22.5	23.3	25.7	2.3	0.005
CA/GA	27.6	25.0	21.9	24.8	1.3	0.008
CY/K3G	31.5	27.6	24.1	27.7	1.7	0.006
CY/GA	26.2	24.1	24.1	24.8	0.6	0.005
K3G/GA	20.8	17.8	21.3	20.0	0.9	0.954

(B) 07124B

Phenolic Treatment	Replicate 1	Replicate 2	Replicate 3	Mean	S.E.M.	p-Value
DMSO	28.1	28.1	30.3	28.8	0.6	
CA	42.3	37.7	34.3	38.1	1.9	0.767
CY	41.2	55.8	117.8	71.6	19.2	0.317
K3G	31.0	23.9	33.3	29.4	2.3	1.000
GA	19.7	28.4	28.7	25.6	2.4	0.468
All 4	27.1	28.1	39.2	31.5	3.2	1.000
CA/CY	36.2	61.9	46.1	48.1	6.1	0.317
CA/K3G	31.5	28.1	35.6	31.7	1.8	1.000
CA/GA	35.5	33.3	31.9	33.6	0.9	0.987
CY/K3G	31.4	43.4	31.9	35.6	3.2	0.965
CY/GA	31.9	44.8	34.4	37.0	3.2	0.898
K3G/GA	28.4	32.8	37.0	32.7	2.0	0.999

Supplemental Table S2. Phenolic treatment impacts on *SIRT1* transcript abundance. Provided are the mean values of three independent biological replicates (n = 3) that were normalized to the DMSO control.

Phenolic Treatment	2DD mean fold change (± SEM)	07124B mean fold change (± SEM)
DMSO	1.0	1.0
CA	1.2 ± 0.3	-1.0 ± 0.1
CY	-1.2 ± 0.2	-1.1 ± 0.1

K3G	1.6 ± 0.4	1.2 ± 0.1
GA	-1.1 ± 0.1	-1.0 ± 0.1
All 4	-1.2 ± 0.1	-1.2 ± 0.1
CA/CY	1.1 ± 0.2	1.4 ± 0.2
CA/K3G	1.0 ± 0.2	1.5 ± 0.3
CA/GA	1.1 ± 0.2	-1.0 ± 0.1
CY/K3G	-1.0 ± 0.3	1.1 ± 0.1
CY/GA	1.1 ± 0.1	1.5 ± 0.1
K3G/GA	-1.1 ± 0.2	1.1 ± 0.2

Supplemental Table S3. Phenolic treatment impacts on SIRT1 protein abundance. Values provided are the ratios to control, which were calculated by normalizing the quantified value to the load control and then dividing the treatment by the control value. Three independent biological replicates (*n* = 3) were performed per cell line and experimental condition.

(A) 2DD

Sample	Replicate 1	Replicate 2	Replicate 3	Mean	S.E.M.	*p*-Value
DMSO	1.0	1.0	1.0	1.0	0	
CA	0.8	1.0	0.7	0.8	0.06	0.743
CY	0.7	0.7	0.9	0.8	0.04	0.596
K3G	1.0	1.0	1.2	1.1	0.07	1.000
GA	1.0	1.0	1.5	1.2	0.2	0.876
All 4	0.8	0.8	0.8	0.8	0.02	0.553
CA/CY	0.6	0.8	0.7	0.7	0.04	0.111
CA/K3G	0.8	0.8	0.8	0.8	0.009	0.447
CA/GA	0.7	0.7	0.8	0.8	0.03	0.406
CY/K3G	0.7	0.8	0.7	0.7	0.02	0.185
CY/GA	0.7	0.7	0.6	0.7	0.02	0.065
K3G/GA	0.9	0.8	0.8	0.9	0.03	0.296

(B) 07124B

Sample	Replicate 1	Replicate 2	Replicate 3	Mean	S.E.M.	*p*-Value
DMSO	1.0	1.0	1.0	1.0	0	
CA	0.9	0.9	1.0	0.9	0.02	0.994
CY	0.9	0.8	1.0	0.9	0.03	0.767
K3G	0.9	1.0	1.1	1.0	0.05	1.000
GA	0.8	0.9	1.2	1.0	0.08	1.000
All 4	0.8	0.8	1.0	0.9	0.07	0.494
CA/CY	0.8	0.7	1.0	0.8	0.06	0.150
CA/K3G	0.8	0.7	1.0	0.9	0.07	0.586
CA/GA	0.8	0.7	1.0	0.8	0.05	0.274
CY/K3G	0.8	0.7	0.8	0.8	0.04	0.049
CY/GA	0.8	0.7	0.8	0.8	0.03	0.074

K3G/GA	1.0	0.7	0.9	0.9	0.07	0.776

(C) 2DD

Sample	Replicate 1	Replicate 2	Replicate 3	Mean	S.E.M.	*p*-Value
DMSO	1.0	1.0	1.0	1.0	0	
DMSO H_2O_2	1.0	1.2	0.4	0.9	0.2	0.997
CA H_2O_2	0.9	0.6	0.1	0.6	0.2	0.152
CY H_2O_2	0.8	0.4	0.1	0.4	0.2	0.025
K3G H_2O_2	0.9	0.9	0.8	0.9	0.01	0.999
GA H_2O_2	0.9	0.8	0.6	0.7	0.1	0.781
All 4 H_2O_2	0.8	0.2	0.2	0.4	0.1	0.021
CA/CY H_2O_2	0.8	0.2	0.2	0.4	0.2	0.009
CA/K3G H_2O_2	0.9	0.5	0.4	0.6	0.1	0.159
CA/GA H_2O_2	0.9	0.7	0.2	0.6	0.2	0.183
CY/K3G H_2O_2	0.9	0.4	0.1	0.5	0.2	0.035
CY/GA H_2O_2	0.9	0.3	0.1	0.4	0.2	0.021
K3G/GA H_2O_2	1.0	0.8	0.5	0.7	0.1	0.825

(D) 07124B

Sample	Replicate 1	Replicate 2	Replicate 3	Mean	S.E.M.	*p*-Value
DMSO	1.0	1.0	1.0	1.0	0	
DMSO H_2O_2	1.0	0.8	1.1	1.0	0.1	1.000
CA H_2O_2	1.0	0.8	0.2	0.7	0.3	0.926
CY H_2O_2	1.0	0.8	0.2	0.6	0.3	0.896
K3G H_2O_2	1.0	1.7	1.1	1.3	0.3	0.981
GA H_2O_2	1.0	1.0	1.6	1.2	0.3	0.999
All 4	0.9	0.5	0.4	0.6	0.2	0.878

H_2O_2						
CA/CY H_2O_2	0.9	0.4	0.2	0.5	0.3	0.601
CA/K3G H_2O_2	0.9	0.4	0.5	0.6	0.2	0.876
CA/GA H_2O_2	1.0	0.5	0.6	0.7	0.2	0.972
CY/K3G H_2O_2	0.9	0.5	0.4	0.6	0.2	0.802
CY/GA H_2O_2	1.0	0.3	0.4	0.6	0.3	0.730
K3G/GA H_2O_2	1.0	0.5	1.0	0.8	0.2	1.000

Supplemental Table S4. Phenolic treatment impact on SIRT1 activity level normalized to control.

(A) 2DD

Treatment	Replicate 1	Replicate 2	Average	S.E.M	*p*-Value
DMSO	1.0	1.0	1.0	0	
CA	1.5	1.6	1.5	0.05	0.001
CY	1.5	1.4	1.5	0.03	0.003
K3G	1.2	1.4	1.3	0.04	0.365
GA	1.0	1.5	1.3	0.2	0.519
All 4	1.0	1.3	1.1	0.1	0.999
CA/CY	0.9	1.2	1.0	0.1	1.000
CA/K3G	1.1	1.3	1.3	0.1	0.936
CA/GA	0.8	1.2	1.0	0.1	1.000
CY/K3G	1.1	1.7	1.4	0.2	0.051
CY/GA	1.2	1.6	1.4	0.2	0.060
K3G/GA	1.0	1.6	1.3	0.2	0.319

(B) 07124B

Treatment	Replicate 1	Replicate 2	Average	S.E.M	*p*-Value
DMSO	1.0	1.0	1.0	0	
CA	0.9	1.0	1.0	0.05	1.000
CY	1.4	1.1	1.2	0.1	0.196
K3G	1.0	1.2	1.0	0.001	1.000
GA	1.2	1.1	1.2	0.01	0.663
All 4	1.2	1.1	1.1	0.03	0.908
CA/CY	1.4	1.1	1.2	0.1	0.184
CA/K3G	1.2	1.0	1.1	0.1	0.998
CA/GA	1.2	0.9	1.0	0.1	1.000
CY/K3G	1.1	0.9	1.0	0.1	1.000
CY/GA	1.2	1.0	1.1	0.1	0.957

K3G/GA	1.0	0.8	0.9	0.1	0.998

Supplemental Figure S1. Representative western blot with 07124B cells illustrating antibody specificity. Per well, 10 µg of protein was loaded, nitrocellulose membrane used, primary and secondary antibodies were diluted 1:1000.

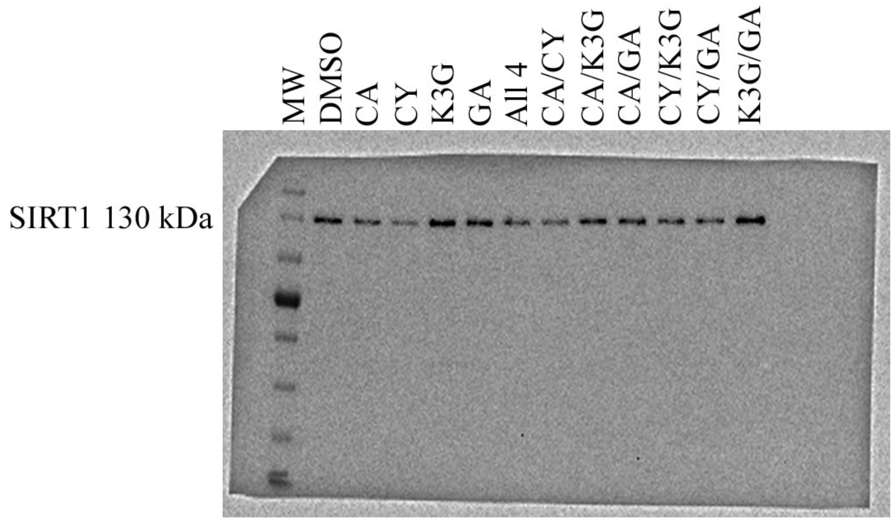

Supplement: Supplementary file 1 [file cells-14-00295-s001.zip › cells-3456963-supplementary.pdf]
